# Long-term Psychological Effects of the COVID-19 Pandemic on Patients with Alcohol Use Disorder

**DOI:** 10.1192/j.eurpsy.2022.528

**Published:** 2022-09-01

**Authors:** I. Fuchs-Leitner, J. Rosenleitner, N. Gerstgrasser, K. Yazdi

**Affiliations:** Kepler University Hospital Linz, Department Of Psychiatry - Specialization Addiction Medicine, Linz, Austria

**Keywords:** alcohol use disorder (AUD), Covid-19, PTSD, DASS-21

## Abstract

**Introduction:**

Vulnerable groups like patients suffering from alcohol use disorders (AUD) are expected to be particularly affected by the negative consequences of the COVID-19 pandemic. In a prior study (N=127), we found that psychosocial COVID-19 factors and living alone elevated the probability for relapse during the initial stage of the pandemic, whereas long-term effects on mental health have yet to be investigated.

**Objectives:**

Here we aimed to investigate the risk of PTSD, as well as levels and developments in depression, anxiety and stress symptomatology as a consequence of the COVID-19 pandemic among patients with AUD.

**Methods:**

Data was collected from a clinical sample of patients with AUD (N=136) in late 2020 and early 2021. PTSD symptoms due to the pandemic were assessed using an adapted version of the impact of event scale (IES-R). Levels in clinical symptoms were collected on the depression, anxiety and stress scale (DASS-21), and changes since the onset of the pandemic were assessed additionally.

**Results:**

The high-risk PTSD-group showed higher levels of depression, anxiety and stress, and reported more severe deteriorations in these symptoms, when compared to the low-risk group. A binary logistic regression model revealed psychological and social aspects of the pandemic to increase the probability for PTSD, whereas sociodemographic and other COVID-19 related factors showed no significant effects.

**Conclusions:**

About 30% of patients with AUD indicated an elevated risk of PTSD due to the pandemic, as well as deteriorations in levels of depression, anxiety and stress. These concerning findings should be especially considered in current and future treatment settings.

**Disclosure:**

No significant relationships.

